# Atherosclerosis: A Pathologist’s Perspective

**DOI:** 10.3390/jcdd13020085

**Published:** 2026-02-09

**Authors:** Ludmila Verboova, Adam Nedoroscik, Terezia Kiskova-Simkova, Adriana Smirjakova, Peter Bohus, Marek Kollar, Michal Virag, Kristína Mazarova, Martina Zavacka

**Affiliations:** 1 Department of Pathology, Faculty of Medicine, University of Pavol Jozef Šafárik in Košice, Rastislavova 43, 040 11 Košice, Slovakia; ludmila.verboova@upjs.sk (L.V.); terezia.kiskova@upjs.sk (T.K.-S.); adriana.smirjakova@student.upjs.sk (A.S.); peter.bohus@upjs.sk (P.B.); 2 The Clinical and Transplant Pathology Centre, Institute of Clinical and Experimental Medicine, 140 21 Prague, Czech Republic; marek.kollar@ikem.cz; 3 Department of Vascular Surgery, Faculty of Medicine, East Slovak Institute of Cardiovascular Diseases, University of Pavol Jozef Šafárik in Košice, 040 01 Košice, Slovakiamartina.zavacka@upjs.sk (M.Z.)

**Keywords:** atherosclerosis, pathology, plaque instability, immunohistochemistry, risk assessment, prevention, therapeutic interventions

## Abstract

Atherosclerosis is a chronic, progressive disease of the arterial wall and the principal pathological substrate underlying most cardiovascular diseases, including ischemic heart disease, stroke, and peripheral arterial disease. Despite advances in prevention, imaging, and therapy, atherosclerosis remains the leading cause of cardiovascular morbidity and mortality worldwide. From a pathological perspective, the disease represents a dynamic and heterogeneous process characterized by endothelial dysfunction, lipid retention and modification, chronic inflammation, immune activation, smooth muscle cell phenotypic modulation, extracellular matrix remodeling, and thrombogenic surface alterations. This review provides a comprehensive overview of atherosclerosis from a pathologist’s perspective, integrating classical morphological concepts with contemporary insights into immunopathology, plaque classification, and mechanisms of plaque instability. We summarize the structure and function of the arterial wall, the stepwise pathogenesis of lesion initiation and progression, and the histopathological classification systems established by the American Heart Association and subsequently refined through Virmani’s framework. Particular emphasis is placed on plaque instability, highlighting the qualitative features—such as fibrous cap thinning, necrotic core expansion, macrophage-driven inflammation, plaque erosion, and calcification patterns—that determine clinical outcomes rather than luminal stenosis alone. Furthermore, the review discusses the expanding role of immunohistochemical markers in defining plaque biology, including lineage markers and functional indicators of inflammation, matrix integrity, osteogenic signaling, and local anticoagulant balance. These pathological insights are integrated with contemporary risk assessment tools, imaging modalities, preventive strategies, and therapeutic interventions, including emerging lipid-lowering and RNA-based therapies. In conclusion, pathology remains central to understanding atherosclerosis as a biologically active disease and to refining concepts of plaque instability. Integrating histopathology with molecular profiling, imaging, and clinical data is essential for advancing precision prevention and targeted treatment strategies in atherosclerotic cardiovascular disease.

## 1. Introduction

Atherosclerosis is a chronic, progressive disease of the arterial wall and the principal pathological substrate underlying most cardiovascular diseases (CVDs), including ischemic heart disease, stroke, and peripheral arterial disease. Despite substantial advances in prevention, diagnosis, and therapy, atherosclerosis remains the leading cause of morbidity and mortality worldwide, accounting for the majority of cardiovascular deaths across diverse populations [1,2]. Its global impact reflects not only the widespread prevalence of traditional risk factors—such as dyslipidemia, hypertension, diabetes mellitus, and smoking—but also complex interactions between metabolic, inflammatory, and immune-mediated processes within the vascular wall [1,3,4].

From a pathological standpoint, atherosclerosis is best understood as a dynamic and heterogeneous disease rather than a uniform process of luminal narrowing. Early descriptions emphasized lipid accumulation and intimal thickening; however, decades of experimental and human pathological studies have established atherosclerosis as a chronic inflammatory disorder characterized by endothelial dysfunction, lipoprotein retention and modification, immune cell recruitment, smooth muscle cell phenotypic modulation, extracellular matrix remodeling, and, ultimately, plaque destabilization and thrombosis [5,6,7]. These processes evolve over time and differ markedly between vascular beds and individuals, giving rise to plaques with variable morphology, biological activity, and clinical consequences.

Pathology has played a central role in defining the natural history of atherosclerosis. Seminal histopathological classifications introduced by the American Heart Association (AHA) and later refined through studies of sudden coronary death have provided a structured framework for understanding lesion progression from adaptive intimal changes and fatty streaks to fibroatheromas and complicated plaques [8,9,10,11]. Importantly, pathological investigations have demonstrated that acute cardiovascular events are most often triggered not by the largest or most obstructive plaques, but by biologically active lesions with thin fibrous caps, large necrotic cores, intense inflammation, plaque erosion, or calcified nodules—features that may not be apparent from angiographic assessment alone [12,13,14].

In recent years, advances in immunohistochemistry, molecular profiling, and imaging have further expanded the pathologist’s ability to dissect plaque composition and vulnerability. Detailed characterization of macrophage subpopulations, smooth muscle cell phenotypic switching, adaptive immune responses, and extracellular matrix integrity has refined our understanding of plaque stability and rupture mechanisms [15,16,17,18,19]. These insights have direct clinical relevance, informing contemporary concepts of risk stratification, preventive strategies, and emerging targeted therapies aimed at plaque stabilization rather than simple luminal enlargement.

This review provides a comprehensive overview of atherosclerosis from a pathologist’s perspective, integrating classical morphological concepts with modern insights into immunopathology, plaque classification, and markers of instability. By linking histopathological features to clinical outcomes and therapeutic implications, we aim to highlight the continued relevance of pathology in advancing the understanding, diagnosis, and management of atherosclerotic cardiovascular disease.

## 2. Epidemiology

Atherosclerosis underlies the majority of cardiovascular diseases and remains the leading cause of cardiovascular-related mortality worldwide. According to recent reports from the World Health Organization (WHO) cardiovascular diseases accounted for approximately 17.9 million deaths globally in 2019 [2,3,20].

The prevalence of atherosclerosis and its clinical manifestations shows marked geographical and socioeconomic variability, driven by differences in lifestyle, environmental exposures, healthcare access, and the distribution of established cardiovascular risk factors [3,21]. From a pathologist’s perspective, these disparities translate into heterogeneity in plaque burden, composition, and complication rates observed across populations.

In high-income countries, the incidence and mortality of atherosclerotic cardiovascular disease (ASCVD) have declined over recent decades. This favorable trend is largely attributed to advances in preventive and therapeutic strategies, including widespread use of statins and antihypertensive agents, improved acute coronary and stroke care, and effective public health interventions targeting smoking cessation and dietary modification [1,20,21]. These changes have altered both the natural history of atherosclerosis and the pathological spectrum of lesions encountered, with increased detection of earlier-stage or medically stabilized plaques.

In contrast, low- and middle-income countries (LMICs) have experienced a sustained rise in atherosclerosis-related morbidity and mortality. This increase reflects an ongoing epidemiological transition, in which non-communicable diseases have overtaken infectious diseases as the leading causes of death [3,22]. LMICs now account for more than three-quarters of global cardiovascular deaths, a disproportionate burden that is mirrored pathologically by higher rates of advanced, unstable, and untreated atherosclerotic lesions [2,3,22].

## 3. Pathology

Understanding the pathology of atherosclerosis is essential for developing therapeutic strategies aimed to prevent and to treat cardiovascular diseases (CVD) [7].

### 3.1. The Arterial Wall: Structure and Function

**Figure 1:**
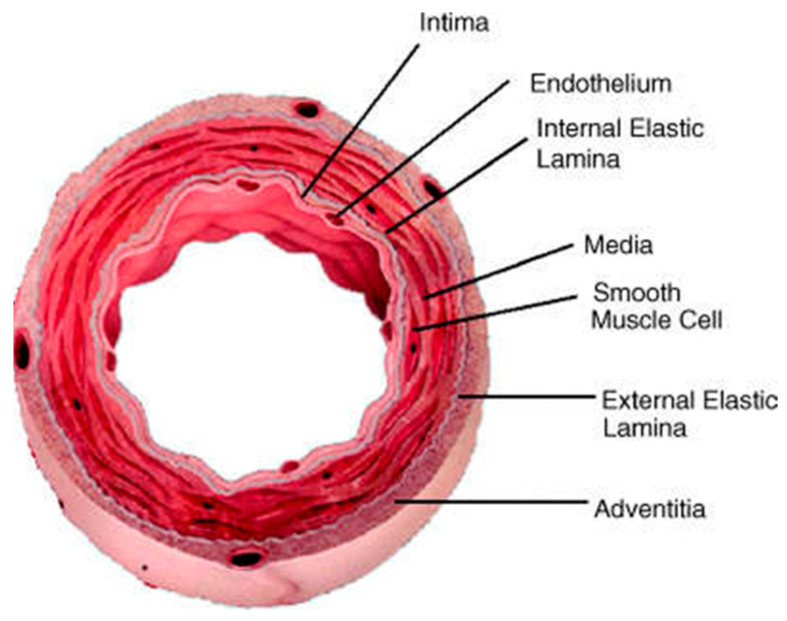
The point of view of the arterial wall comprises three main layers of the Tunica: intima, media, and adventitia. A single layer of endothelial cells covers the lumen, while smooth muscle cells and fibroblast cells comprise the outer layers. Reprinted from ref [23].

The arterial wall consists of three layers: the intima, media, and adventitia (Figure 1). Each plays a crucial role in maintaining vascular homeostasis and responding to pathological stimuli.

- Intima: The innermost layer of the arterial wall, the intima, is composed of a single layer of endothelial cells that line the lumen of the artery. The endothelium serves as a barrier between the blood and the underlying tissues, regulating vascular tone, blood flow, and the exchange of nutrients and waste products. It also plays a crucial role in preventing thrombosis by producing antithrombotic molecules such as nitric oxide (NO) and prostacyclin [23].
- Media: The media is the middle layer of the arterial wall and consists of smooth muscle cells (SMCs) embedded in an extracellular matrix (ECM) composed of collagen, elastin, and proteoglycans. The media is responsible for the structural integrity and elasticity of the artery, allowing it to withstand and respond to changes in blood pressure [24].
- Adventitia: The outermost layer of the arterial wall, the adventitia, contains fibroblasts, collagen fibers, nerves, and the vasa vasorum (small blood vessels that supply nutrients to the arterial wall). The adventitia provides structural support and plays a role in inflammatory response by serving as a site for immune cell accumulation and activation [25].

### 3.2. Pathogenesis of Atherosclerosis

#### 3.2.1. Initiation of Atherosclerosis

Atherosclerosis begins with endothelial dysfunction, a condition in which the normal protective functions of the endothelium are compromised. This dysfunction is triggered by various risk factors, including hyperlipidemia, hypertension, smoking, diabetes, and chronic inflammation [26].

Atherosclerosis is characterized by the accumulation of lipids and fibrous tissue within large and medium-sized arteries. Very low-density lipoproteins (VLDLs), synthesized by hepatocytes, are released into the circulation and undergo progressive lipolysis, giving rise to intermediate-density lipoproteins and ultimately low-density lipoproteins (LDLs). LDL particles are the principal carriers of cholesterol in the bloodstream and play a central role in atherogenesis by infiltrating the tunica intima through the arterial endothelium. Under physiological conditions, the intact endothelium limits LDL entry; however, endothelial dysfunction caused by turbulent flow, mechanical stress, or inflammation increases vascular permeability, facilitating LDL retention and subsequent plaque formation [26].

a. Endothelial Dysfunction

The onset of atherosclerosis involves endothelial cell activation and dysfunction. Endothelial dysfunction is associated with reduced NO bioavailability, elevated reactive oxygen species (ROS) production, and enhanced expression of adhesion molecules such as vascular cell adhesion molecule-1 (VCAM-1), intercellular adhesion molecule-1 (ICAM-1), and E-selectin. These alterations facilitate the recruitment and adhesion of circulating leukocytes, especially monocytes, to the endothelial surface [6].

b. Lipoprotein Retention and Modification

Low-density lipoprotein (LDL) particles penetrate the endothelial barrier and accumulate in the intima, undergoing oxidative modification by ROS and enzymes such as myeloperoxidase and lipoxygenase. Oxidized LDL (oxLDL) is highly atherogenic, and may contribute to the onset of atherosclerosis by promoting inflammation, endothelial dysfunction, and the recruitment of immune cells [27].

c. Monocyte Recruitment and Differentiation

Monocytes adhere to activated endothelium via interactions with adhesion molecules and chemokines, including monocyte chemoattractant protein-1 (MCP-1). Once in the intima, monocytes differentiate into macrophages, which are key players in the development of atherosclerotic lesions. Macrophages internalize modified low-density lipoproteins, including oxidized LDL (oxLDL), minimally modified LDL, and glycated LDL, through scavenger receptors such as CD36 and SR-A, leading to the formation of lipid-laden foam cells [28].

#### 3.2.2. Progression of Atherosclerosis

**Figure 2:**
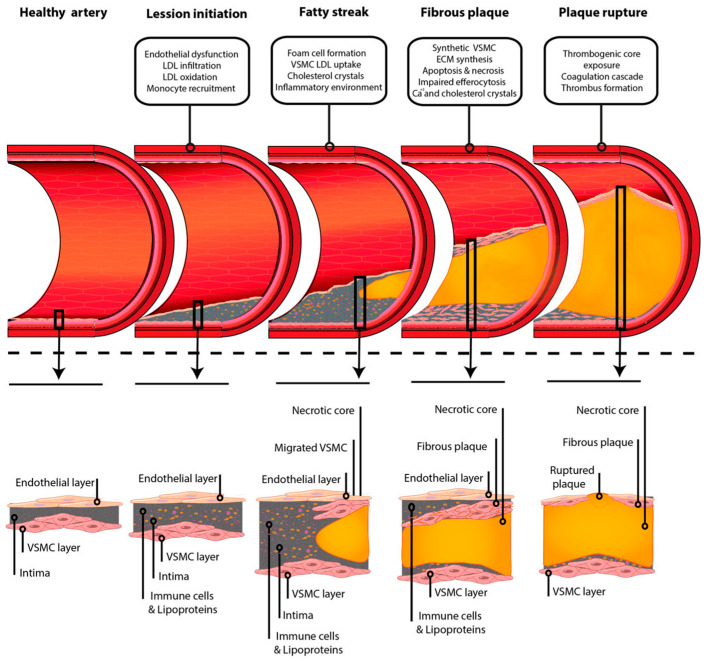
Schematic representation of atheroma plaque formation from a healthy artery to plaque rupture underlying the most important events that contribute to its development in each stage. Reprinted from ref [28].

The progression of atherosclerosis is characterized by the formation of atherosclerotic plaques, consisting of a lipid core, a fibrous cap, and various inflammatory cells. As plaques grow and mature, they can become unstable and prone to rupture, leading to clinical complications (Figure 2) [29].

a. Foam Cell Formation and Lipid Accumulation

Foam cells, primarily derived from macrophages and, to a lesser extent, smooth muscle cells (SMCs), are a hallmark of early atherosclerotic lesions, known as fatty streaks. These cells accumulate substantial amounts of lipids, mainly cholesterol esters, and secrete pro-inflammatory cytokines and chemokines, sustaining the inflammatory response. The ongoing recruitment of monocytes and foam cell accumulation contribute to the enlargement of the lipid core [13,30].

b. Smooth Muscle Cell Migration and Proliferation

SMCs from the media migrate into the intima in response to growth factors such as platelet-derived growth factor (PDGF) and transforming growth factor-beta (TGF-β). In the intima, SMCs undergo phenotypic modulation from a contractile to a synthetic phenotype, characterized by increased proliferation, migration, and ECM production. SMCs synthesize collagen, elastin, and proteoglycans, contributing to the formation of the fibrous cap, which overlies the lipid core and provides structural stability to the plaque [10].

c. Extracellular Matrix Remodeling

ECM remodeling plays a crucial role in plaque stability. Matrix metalloproteinases (MMPs), secreted by macrophages, SMCs, and other cells, degrade ECM components such as collagen and elastin, weakening the fibrous cap and increasing the risk of plaque rupture. In contrast, tissue inhibitors of metalloproteinases (TIMPs) counteract MMP activity and promote ECM deposition, contributing to plaque stabilization [31].

d. Neovascularization and Intraplaque Hemorrhage

As atherosclerotic plaques progress, they may develop neovessels from the vasa vasorum, a process known as neovascularization. These new, fragile vessels are leaky, causing red blood cells and inflammatory cells to leak into the plaque. Intraplaque hemorrhage contributes to the rapid progression of advanced lesions and is associated with an increased risk of clinical events

#### 3.2.3. Plaque Destabilization and Complications

The stability of the atherosclerotic plaque largely determines the clinical consequences of atherosclerosis. Plaque destabilization, characterized by thinning of the fibrous cap, increased lipid content, and heightened inflammatory activity, can lead to plaque rupture, thrombosis, and acute cardiovascular events [12].

a. Plaque Rupture

Plaque rupture occurs when the fibrous cap becomes thin and weak, typically due to extensive ECM degradation by MMPs and reduced collagen synthesis by SMCs [32]. Rupture exposes the highly thrombogenic lipid core to the bloodstream, triggering the formation of a thrombus (blood clot). Thrombus formation can lead to the occlusion of the artery and result in acute myocardial infarction (heart attack) or ischemic stroke, depending on the location of the affected artery [12].

b. Plaque Erosion

In addition to plaque rupture, plaque erosion represents another mechanism underlying cardiovascular events. It occurs when the endothelial layer covering the plaque becomes disrupted, exposing the subendothelial matrix to circulating platelets and facilitating thrombus formation. Plaque erosion is seen more frequently in younger individuals and in women, and it is associated with risk factors that differ from those linked to plaque rupture [4].

c. Calcification and Stiffening

As plaques progress, they may undergo calcification, a process mediated by SMCs and macrophages that resemble bone formation [4]. Calcification can stabilize plaques by increasing their rigidity, but it may also lead to arterial stiffening, reduced vessel compliance, and increased risk of hypertension and left ventricular hypertrophy [4].

### 3.3. Classification of Atherosclerotic Lesion

Early pathological characterizations of atherosclerosis described a continuum of lesion development ranging from fatty streaks to fibroatheromas (FAs) and ultimately to advanced plaques complicated by intraplaque hemorrhage, calcification, surface ulceration, and thrombosis [3,4,33]. To standardize lesion nomenclature and improve clinicopathologic correlations, the American Heart Association (AHA) Consensus Group led by Herbert C. Stary introduced a refined histopathological classification in the mid-1990s that remains foundational in atherosclerosis research and continues to underpin contemporary pathological and imaging studies [11,34].

The AHA classification defines six principal lesion categories. Early or precursor lesions include type I lesions (initial lesions or adaptive intimal thickening), type II lesions (fatty streaks or intimal xanthomas), and type III lesions (intermediate or transitional lesions characterized by early extracellular lipid accumulation). Advanced atherosclerotic plaques are classified as type IV lesions (atheromas with a well-formed necrotic lipid core), type V lesions (fibroatheromas with a thick fibrous cap or predominantly fibrotic plaques), and type VI lesions (complicated plaques), which are defined by surface disruption, intraplaque hematoma or hemorrhage, and/or superimposed luminal thrombosis [8,9,11,34].

**Figure 3:**
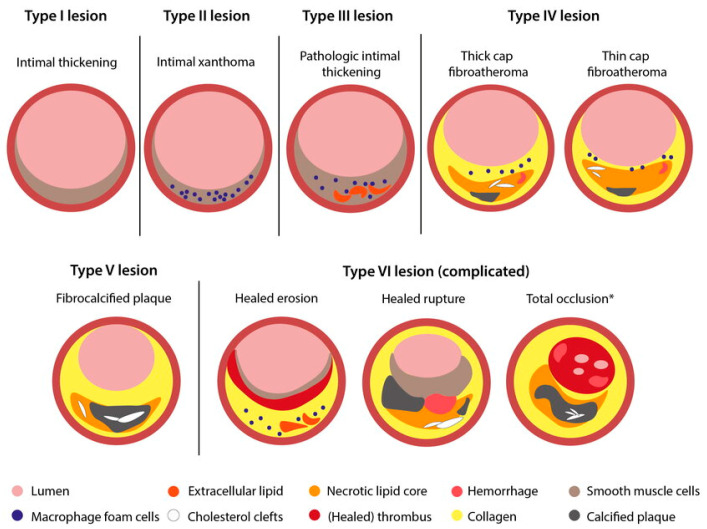
Modified AHA classification of atherosclerotic lesions. Reprinted from ref [35].

Subsequent refinements of the AHA scheme, largely driven by the seminal pathological studies of sudden coronary death by Virmani and colleagues, expanded the classification to incorporate lesion phenotypes responsible for acute thrombosis independent of classic plaque rupture. This Virmani-modified framework replaced numeric AHA lesion types I–IV with descriptive terminology, including adaptive intimal thickening, intimal xanthoma, pathological intimal thickening (PIT), and fibroatheroma, and explicitly recognized plaque erosion and calcified nodules as distinct high-risk substrates of coronary thrombosis (Figure 3) [8,9,10,14]. These concepts are now widely integrated into modern atherosclerosis pathology, intravascular imaging interpretation, and translational research focused on plaque instability and clinical risk stratification (Table 1) [3,10,33].

Development of human coronary atherosclerosis. The two nonprogressive lesions are intimal thickening or intimal xanthoma (foam cell collections known as fatty streaks, AHA Type II). Pathological intimal thickening (AHA Type III, transitional lesions) is the first of progressive plaques marked by an acellular lipid pool rich in proteoglycan; inflammation when present is typically confined to the most luminal aspect of this plaque. Fibroatheromas are lesions with areas of necrosis characterized by cellular debris and cholesterol monohydrate with varying degrees of calcification or hemorrhage. Finally, thin-cap fibroatheroma or vulnerable plaques are recognized by their relatively large necrotic cores and thin fibrous caps.

## 4. Immunohistochemical Markers in Atherosclerotic Plaques

Immunohistochemical assessment of atherosclerotic plaques begins with identification of the principal cellular lineages that define lesion composition and architecture. Establishing this cellular framework is essential for interpreting downstream processes related to inflammation, progression, and plaque instability.

### 4.1. Core Lineage Panels

From a pathology perspective, defining “who populates the atherosclerotic plaque” is the first essential step toward understanding plaque behavior. Core lineage immunohistochemical (IHC) panels identify the cellular composition and spatial organization of plaques, providing the biological context within which instability, progression, and thrombosis occur. While no single lineage marker predicts vulnerability in isolation, the relative abundance, localization, and interaction of immune and vascular cell populations strongly influence plaque fate [3,11,33].

#### 4.1.1. Macrophages and Foam Cells (CD68)

CD68 remains the most widely used pan-macrophage marker in human atherosclerotic tissue and is central to any core lineage panel. CD68 highlights macrophages and macrophage-derived foam cells throughout the plaque, with particular diagnostic relevance in the shoulder regions, fibrous cap–adjacent areas, and necrotic core interface.

From an instability standpoint, increased CD68^+^ macrophage density in the fibrous cap and shoulders correlates with cap thinning, matrix degradation, and heightened risk of rupture. Pathologically, it is not the total macrophage burden but the focal accumulation at mechanically stressed regions that best reflects vulnerability. Consequently, CD68 quantification is often performed semi-quantitatively within defined regions of interest rather than across the entire plaque [33,36,37].

#### 4.1.2. Smooth Muscle Cells and Cap Integrity (α-SMA)

α-smooth muscle actin (α-SMA) identifies vascular smooth muscle cells (SMCs) and myofibroblast-like cells and is indispensable for assessing fibrous cap structure and stability. In stable plaques, α-SMA–positive SMCs form a dense, collagen-producing cap that confers mechanical strength and resistance to rupture.

Loss or redistribution of α-SMA staining within the cap—particularly when accompanied by increased macrophage infiltration—signals cap weakening and phenotypic modulation of SMCs, a key process in plaque destabilization. From a pathologist’s perspective, juxtaposition of α-SMA and CD68 staining provides a powerful visual representation of the balance between repair (SMCs) and injury (macrophage-mediated inflammation) [11,15,16].

#### 4.1.3. T Lymphocytes (CD3 and CD8)

CD3 identifies the total T-cell population within plaques and is commonly used to assess the contribution of adaptive immunity to plaque biology. T cells are typically concentrated in the adventitia, plaque shoulders, and fibrous cap, where they modulate macrophage and SMC behavior through cytokine signaling.

Subtyping with CD8 highlights cytotoxic T lymphocytes, which are increasingly recognized as contributors to plaque instability. CD8^+^ T cells localize to areas of active inflammation and may promote macrophage activation, endothelial dysfunction, and SMC apoptosis, thereby indirectly weakening the fibrous cap. An increased CD8^+^/CD3^+^ ratio within cap or shoulder regions has been associated with advanced and unstable plaque phenotypes [4,17,18].

#### 4.1.4. B Lymphocytes (CD20)

CD20 marks B lymphocytes, which are less abundant than macrophages or T cells but play an important modulatory role in atherosclerosis. CD20^+^ cells are most commonly identified in the adventitia and perivascular regions, often forming organized aggregates reminiscent of tertiary lymphoid structures.

From a vulnerability perspective, B cells may exert both protective and pro-atherogenic effects, depending on subset and context. Although routine IHC does not distinguish B-cell subtypes, the presence and organization of CD20^+^ cells provide insight into chronic immune activation and plaque chronicity, which may influence long-term progression rather than acute rupture.

From a pathologist’s perspective, core lineage immunohistochemical markers define the cellular ecosystem of the atherosclerotic plaque. The relative abundance and spatial organization of macrophages (CD68), smooth muscle cells (α-SMA), and adaptive immune populations (CD3, CD8, CD20) provide critical context for interpreting plaque stability versus vulnerability [4,38].

### 4.2. Immunohistochemical Markers of Plaque Instability

From a pathologist’s perspective, plaque instability reflects biological activity within the lesion that predisposes to surface disruption, thrombosis, or rapid progression, rather than plaque size alone. While traditional immunohistochemical (IHC) panels identify major cellular components (macrophages, smooth muscle cells, endothelium), increasing attention has focused on functional markers that report macrophage activation states, extracellular matrix remodeling, and osteogenic signaling. These markers provide insight into mechanisms of instability, including necrotic core expansion, fibrous cap weakening, maladaptive calcification and in general provide complementary information on plaque homeostasis versus vulnerability.

#### 4.2.1. TREM2: Macrophage Phenotype and Lipid-Driven Instability

Triggering receptor expressed on myeloid cells-2 (TREM2) has emerged as a key marker of a distinct macrophage population within advanced atherosclerotic plaques. TREM2-positive macrophages are enriched in lipid-rich, necrotic core–adjacent regions and are strongly associated with foam cell biology, defective efferocytosis, and altered inflammatory signaling. From a pathological standpoint, TREM2 expression identifies macrophages that are metabolically adapted to lipid overload and necrotic debris. Recent human plaque studies integrating transcriptomics with immunohistochemistry demonstrate that TREM2^+^ macrophages localize preferentially to unstable plaques, particularly those with large necrotic cores and thin fibrous caps. These cells appear less effective at resolving inflammation, thereby contributing to chronic inflammatory stress and cap vulnerability [17,39].

IHC detection of TREM2, often in combination with CD68 or CD163, is therefore increasingly used to refine macrophage heterogeneity beyond simple density measures, linking macrophage quality rather than quantity to plaque instability [40].

#### 4.2.2. Mimecan (Osteoglycin): Extracellular Matrix Organization and Cap Integrity

Mimecan (osteoglycin) is a small leucine-rich proteoglycan involved in collagen fibrillogenesis and extracellular matrix organization. In the context of atherosclerosis, mimecan expression is closely associated with fibrous cap structure and mechanical stability. Pathological studies of human carotid and coronary plaques have demonstrated reduced mimecan expression in unstable lesions, particularly in regions of cap thinning and rupture. Loss or disorganization of mimecan-positive matrix correlates with disrupted collagen architecture, reduced tensile strength, and increased susceptibility to mechanical failure [41,42]

From an IHC perspective, mimecan serves as a matrix-stability marker, complementing smooth muscle cell markers such as αSMA. Reduced mimecan staining in the fibrous cap supports a diagnosis of structural vulnerability, even in plaques without overt rupture [19,42].

#### 4.2.3. Sclerostin: Osteogenic Signaling and Calcification-Associated Vulnerability

Sclerostin, a Wnt signaling antagonist classically associated with bone metabolism, has gained attention in vascular pathology as a marker of osteogenic differentiation and pathological calcification within atherosclerotic plaques. Immunohistochemical studies demonstrate sclerostin expression in calcified regions of advanced plaques, particularly in association with osteoblast-like vascular smooth muscle cells. Importantly, sclerostin expression is not uniformly linked to plaque stability: while large sheet-like calcifications may reflect healed or stable lesions, heterogeneous or nodular calcification, often sclerostin-positive, has been associated with mechanical stress concentration and plaque instability [43,44,45,46].

Thus, sclerostin IHC contributes to the interpretation of calcification quality rather than quantity, helping distinguish potentially stabilizing calcification from calcification patterns associated with rupture or calcified nodules [45,46].

#### 4.2.4. Adiponectin: Metabolic Signaling and Anti-Inflammatory Restraint

Adiponectin, an adipokine classically associated with systemic metabolic regulation, is increasingly recognized within the vascular wall and atherosclerotic plaques. Immunohistochemical studies have localized adiponectin to endothelial cells, smooth muscle cells, and macrophages, with expression patterns varying according to plaque stage and stability. From a pathology perspective, reduced adiponectin expression within plaques correlates with increased inflammatory burden, macrophage activation, and oxidative stress, all hallmarks of vulnerable lesions. Adiponectin exerts anti-inflammatory and anti-atherogenic effects by inhibiting macrophage foam cell formation, suppressing pro-inflammatory cytokine production, and preserving endothelial function. Loss of local adiponectin signaling within the plaque microenvironment may therefore facilitate necrotic core expansion and fibrous cap weakening [47,48,49,50]

In IHC panels, adiponectin serves as a protective counter-regulatory marker, where diminished staining supports a shift toward a pro-instability phenotype, particularly in plaques associated with metabolic dysfunction [49,50].

#### 4.2.5. Adrenomedullin: Endothelial Stress and Compensatory Vasoprotection

Adrenomedullin (ADM) is a vasoactive peptide widely expressed in endothelial cells, vascular smooth muscle cells, and macrophages. Within atherosclerotic plaques, ADM expression is commonly upregulated in response to hypoxia, inflammation, and oxidative stress, conditions frequently encountered in advanced and unstable lesions. Pathologically, increased ADM immunoreactivity is often observed in shoulder regions, neovessels, and macrophage-rich areas, suggesting a compensatory response aimed at maintaining endothelial integrity and limiting inflammatory damage. ADM possesses vasodilatory, anti-apoptotic, and barrier-protective properties; however, persistent upregulation within plaques may also reflect chronic endothelial injury and microvascular dysfunction, both of which are associated with plaque instability and intraplaque hemorrhage [51,52,53,54]

Thus, ADM IHC is best interpreted as a stress-response marker, where heightened expression indicates biologically active, high-risk plaques attempting—but failing—to restore homeostasis [53,54].

#### 4.2.6. Heparin Cofactor II: Local Anticoagulant Balance and Thrombotic Risk

Heparin cofactor II (HCII) is a serine protease inhibitor that selectively inactivates thrombin in the presence of dermatan sulfate, a glycosaminoglycan abundant in the vascular extracellular matrix. Beyond its systemic anticoagulant role, HCII has emerged as an important local modulator of thrombin activity within the arterial wall [55,56,57,58].

Immunohistochemical analyses have demonstrated HCII expression in endothelial cells, smooth muscle cells, and extracellular matrix-rich regions of atherosclerotic plaques. Reduced HCII expression or altered spatial distribution is associated with enhanced thrombin signaling, promoting inflammation, smooth muscle cell dysfunction, and plaque progression [57,58].

From a pathologist’s perspective, immunohistochemical markers of plaque instability increasingly emphasize macrophage functional states (TREM2), matrix integrity (mimecan), and osteogenic signaling (sclerostin). These markers extend beyond cell identification to capture biological processes that underline plaque instability, reinforcing the central role of pathology in validating and refining modern concepts of high-risk atherosclerotic disease. From a pathologist’s perspective, diminished HCII staining within the fibrous cap or plaque surface supports a pro-thrombotic and pro-inflammatory microenvironment, predisposing to plaque-associated thrombosis following rupture or erosion. HCII therefore represents a functional anticoagulant marker, linking matrix biology to thrombotic susceptibility [19,40,42,45,46,47,48,49,50,51,52,53,54,55,56,57,58].

## 5. Risk Assessment Tools

Early detection of atherosclerosis is crucial for preventing its progression and the subsequent development of CVDs. Screening involves identifying individuals at high risk for atherosclerosis before clinical symptoms manifest, enabling timely interventions to mitigate risk and prevent adverse cardiovascular events [7].

### 5.1. Histology-Based Vulnerability Scoring Systems

Several histopathology-driven scoring approaches have been proposed, primarily in research and autopsy cohorts. These systems typically assign weighted scores to individual plaque characteristics, generating a composite “vulnerability index.” Although not universally standardized, common pathological scoring elements include:

(a) Structural instability: thin fibrous cap, cap rupture, or surface erosion;
(b) Inflammatory burden: macrophage density at the cap and shoulder regions;
(c) Thrombogenic substrate: size of necrotic core, tissue factor expression;
(d) Hemorrhagic activity: intraplaque hemorrhage and neovascularization;
(e) Calcification pattern: spotty or nodular calcification associated with stress concentration.

From a pathologist’s standpoint, plaque instability scoring systems represent an evolution from descriptive morphology toward integrated structure–function–biology models of atherosclerosis. While no single scoring system has achieved universal adoption, the principles derived from pathology continue to underpin modern risk stratification and translational research in atherosclerotic disease. Future directions aim to combine quantitative digital pathology, molecular profiling, and biomechanical modeling to refine vulnerability assessment. From a pathology perspective, the ultimate goal is not merely to label plaques as “vulnerable,” but to define biologically actionable phenotypes that inform prevention and targeted therapy [14,36,59].

### 5.2. Imaging Techniques and Risk Assessment Tools

Imaging techniques play a critical role in directly assessing atherosclerotic plaque burden and vascular health. Two of the most used non-invasive imaging modalities for screening are:

- Carotid Intima-Media Thickness (CIMT): CIMT measurement using ultrasound assesses the thickness of the carotid artery walls, which correlates with the extent of atherosclerosis in other vascular beds. Increased CIMT is associated with a higher risk of future cardiovascular events. However, the routine use of CIMT for screening in asymptomatic individuals remains controversial due to variability in measurement and interpretation [60].
- Coronary Artery Calcium (CAC) Scoring: CAC scoring, obtained through non-contrast cardiac computed tomography (CT), quantifies the extent of calcified atherosclerotic plaques in the coronary arteries. The CAC score is a strong predictor of future coronary events and is particularly useful in individuals with intermediate risk, where it can help refine risk stratification and guide preventive interventions. High CAC scores are associated with a higher likelihood of significant coronary artery disease and the need for aggressive preventive measures [61].

### 5.3. Blood Biomarkers and Risk Assessment Tools

Blood biomarkers provide additional information about the risk of atherosclerosis and cardiovascular disease. The most measured biomarkers include:

#### 5.3.1. Lipid Profile

Measuring total cholesterol, LDL, HDL, and triglycerides is a routine part of cardiovascular risk assessment. Elevated LDL is a primary target for intervention in the prevention of atherosclerosis, while low HDL and high triglycerides are also associated with increased risk [27].

#### 5.3.2. High-Sensitivity C-Reactive Protein (hs-CRP)

Hs-CRP is a marker of systemic inflammation and has been shown to be an independent predictor of cardiovascular events. Elevated hs-CRP levels are associated with increased atherosclerotic burden and can help identify individuals at higher risk, particularly when combined with traditional risk factors [62].

#### 5.3.3. Other Biomarkers

Emerging biomarkers such as lipoprotein(a) [Lp(a)], apolipoprotein B (ApoB), and homocysteine are also being investigated for their potential role in improving risk prediction. However, their routine use in clinical practice remains limited due to variability in assay standardization and the need for further validation [63].

### 5.4. Non-Invasive Clinical Risk Assessment Tools

Structural changes are preceded and accompanied by functional disturbances—most notably endothelial dysfunction and arterial stiffening—which can be detected non-invasively before advanced plaque formation. Clinical vascular function tests therefore provide a physiological correlate to the microscopic processes observed in histopathology and serve as early indicators of systemic atherosclerotic disease [64].

#### 5.4.1. Flow-Mediated Vasodilation (FMD)

Flow-mediated vasodilation (FMD) of the brachial artery is a widely used, ultrasound-based technique that assesses endothelium-dependent vasodilation, primarily mediated by nitric oxide (NO). Reduced FMD reflects endothelial dysfunction, a key initiating event in atherogenesis [65].

From a pathological perspective, impaired FMD corresponds to early arterial wall changes, including endothelial activation, increased permeability to lipoproteins, leukocyte adhesion, and reduced antithrombotic capacity. These processes precede the formation of fatty streaks and are not necessarily associated with overt plaque detectable by imaging or histology [4,64].

Importantly, endothelial dysfunction assessed by FMD is systemic, mirroring the diffuse nature of atherosclerosis. Clinical studies demonstrate that reduced FMD predicts future cardiovascular events independently of traditional risk factors, supporting its role as a functional biomarker of early vascular disease. For pathologists, FMD provides a non-invasive window into endothelial biology that complements histological markers such as endothelial denudation, reduced eNOS expression, and inflammatory activation [4,64].

#### 5.4.2. Pulse Wave Velocity (PWV)

Pulse wave velocity (PWV) is the gold-standard non-invasive measure of arterial stiffness, most commonly assessed as carotid–femoral PWV. Increased PWV reflects reduced arterial compliance due to structural remodeling of the arterial wall, including collagen accumulation, elastin fragmentation, smooth muscle cell phenotypic changes, and medial calcification [66].

Pathologically, elevated PWV correlates with advanced atherosclerotic burden and arteriosclerosis, particularly involving the media. Unlike FMD, which captures early functional alterations, PWV reflects cumulative, largely irreversible structural damage, aligning more closely with histological findings such as fibrosis, calcification, and loss of elastic lamellae [66,67].

From a vulnerability standpoint, arterial stiffening increases pulsatile mechanical stress on plaques, particularly at branch points, potentially promoting fibrous cap fatigue and rupture. Thus, PWV integrates both arteriosclerotic remodeling and atherosclerotic risk, bridging large-artery pathology with plaque-level consequences [68].

## 6. Prevention of Atherosclerosis

Preventing atherosclerosis requires a combination of lifestyle modifications, pharmacological interventions, and public health strategies designed to reduce the burden of risk factors associated with the disease.

### 6.1. Lifestyle Modifications

Lifestyle changes are the cornerstone of atherosclerosis prevention and are recommended for all individuals, regardless of their risk level. Key lifestyle modifications include:

#### 6.1.1. Dietary Interventions

A heart-healthy diet is central in preventing atherosclerosis. Diets rich in fruits, vegetables, whole grains, legumes, nuts, and lean proteins, such as the Mediterranean diet, have been shown to reduce the risk of cardiovascular disease. Reducing the intake of saturated fats, trans fats, cholesterol, and refined sugars is also important in lowering LDL levels and preventing the development of atherosclerosis [60].

#### 6.1.2. Physical Activity

Regular physical activity improves cardiovascular health by promoting weight loss, reducing blood pressure, improving lipid profiles, and enhancing insulin sensitivity. Current guidelines recommend at least 150 min of moderate-intensity aerobic exercise or 75 min of vigorous-intensity aerobic exercise per week, combined with muscle-strengthening activities on two or more days per week [69].

#### 6.1.3. Smoking Cessation

Smoking is a major risk factor for atherosclerosis, and cessation is one of the most effective preventive measures. Smoking cessation reduces the risk of cardiovascular events and mortality; the benefits are seen relatively quickly after quitting. Behavioral interventions, nicotine replacement therapy, and pharmacological agents such as varenicline and bupropion are effective strategies for smoking cessation [70].

#### 6.1.4. Weight Management

Obesity is associated with an increased risk of atherosclerosis through its effects on blood pressure, lipid metabolism, and insulin resistance. Achieving and maintaining a healthy weight through diet and physical activity is crucial in reducing cardiovascular risk [70].

## 7. Treatment of Atherosclerosis

### 7.1. Pharmacological Interventions

Pharmacological therapy and lifestyle modifications are often required to achieve optimal risk reduction, particularly in individuals with elevated risk factors or established atherosclerotic disease [71].

#### 7.1.1. Lipid-Lowering Therapy

Statins are the first-line therapy for lowering LDL-C and are widely used for both primary and secondary prevention of atherosclerosis. Statins work by inhibiting HMG-CoA (3-hydroxy-3-methylglutaryl-coenzyme A) reductase, the enzyme responsible for cholesterol synthesis in the liver. They have been shown to significantly reduce the risk of major cardiovascular events in high-risk individuals. Other lipid-lowering agents, such as ezetimibe and PCSK9 inhibitors, are used in cases where statin therapy is insufficient or not tolerated [3].

#### 7.1.2. Antihypertensive Therapy

Hypertension is a significant risk factor for atherosclerosis, and controlling blood pressure is essential for preventing cardiovascular events. Antihypertensive medications, including angiotensin-converting enzyme (ACE) inhibitors, angiotensin II receptor blockers (ARBs), calcium channel blockers, and diuretics, are commonly used to achieve blood pressure targets and reduce cardiovascular risk [72].

#### 7.1.3. Antiplatelet Therapy

Antiplatelet agents, such as aspirin, are used for secondary prevention in individuals with established atherosclerosis to reduce the risk of thrombotic events. However, the use of aspirin for primary prevention is more controversial and is generally reserved for individuals at high risk of cardiovascular events, where the benefits outweigh the potential risks of bleeding [70].

Endovascular and surgical options for treating atherosclerosis include several procedures aimed at restoring blood flow in affected arteries. These interventions are typically considered when conservative treatments are insufficient.

### 7.2. Invasive Procedures

In some patients, especially those with advanced or symptomatic atherosclerosis, invasive procedures may be necessary to restore blood flow and prevent cardiovascular events. Amongst the most common procedures are:

#### 7.2.1. Percutaneous Coronary Intervention (PCI)

PCI, commonly known as angioplasty, involves inserting catheter into a narrowed or blocked coronary artery, followed by the inflation of a balloon to widen the artery. A stent (a small mesh tube) is usually placed at the site to keep the artery open. Drug-eluting stents, which release antiproliferative drugs, are often used to reduce the risk of restenosis (re-narrowing of the artery). PCI is indicated for patients with significant coronary artery stenosis, particularly those with acute coronary syndrome.

#### 7.2.2. Coronary Artery Bypass Grafting (CABG)

CABG is a surgical procedure in which a blood vessel from another part of the body (usually the saphenous vein, internal mammary artery, or radial artery) is grafted to bypass a blocked coronary artery. This procedure is typically reserved for patients with multivessel coronary artery disease or those who are not candidates for PCI. CABG improves blood flow to the heart muscle, reducing symptoms and the risk of myocardial infarction.

#### 7.2.3. Carotid Endarterectomy and Stenting

For patients with significant carotid artery stenosis, carotid endarterectomy (CEA) or carotid artery stenting (CAS) may be indicated to reduce the risk of ischemic stroke. CEA involves the surgical removal of atherosclerotic plaque from the carotid artery, while CAS involves the placement of a stent to widen the artery. These procedures are particularly beneficial in patients with symptomatic carotid stenosis or high-grade asymptomatic stenosis.

#### 7.2.4. Treatment of Lower Extremity Peripheral Artery Disease (PAD) and Chronic Limb-Threatening Ischemia (CLTI)

Treatment options consist of endovascular angioplasty, stenting or atherectomy, whereas surgical options are local endarterectomy or lower extremity bypass surgery. Revascularization (endovascular, surgical, or hybrid) is recommended to prevent limb loss in patients with CLTI. The choice between endovascular and surgical revascularization should be made by a multidisciplinary team, considering the patient’s overall condition, anatomical features, and local expertise. For patients with claudication, revascularization can be considered to improve quality of life and functional status in patients with claudication not responsive to medical therapy and structured exercise. The decision to perform revascularization should be made after a period of optimal medical treatment and exercise and discussed in a multidisciplinary setting. A multidisciplinary team approach is emphasized for complex cases, particularly for CLTI and aortic disease. Individualized decision-making is very important, with the need to consider factors such as patient preferences, comorbidities, and anatomical suitability for different interventions. Hybrid approaches, combining open surgical and endovascular techniques, are increasingly adopted for complex thoracoabdominal aortic diseases [73].

#### 7.2.5. Renal Artery Stenosis

Renal hypertension can be treated by endovascular procedures. Renal artery angioplasty without stenting should be considered for patients with hypertension and hemodynamically significant fibromuscular dysplasia. Renal artery angioplasty and stenting may be considered in patients with hemodynamically significant atherosclerotic renal artery stenosis in specific clinical scenarios, such as recurrent heart failure or resistant hypertension [74].

## 8. New Trends in Pathophysiology and Treatment

Recent years have brought a rapid evolution in our understanding of disease mechanisms, driven by advances in molecular biology, systematic medicine, and high-resolution diagnostic technologies. These developments have reshaped traditional views of pathophysiology, revealing complex networks of genetic, immunologic, and environmental interactions that influence disease onset and progression. Alongside these insights, treatment strategies are shifting toward precision medicine, targeted therapies, and regenerative approaches that aim not only to manage symptoms but also to correct underlying biological dysfunctions. Together, these advances herald a new era in which mechanistic understanding increasingly guides individualized therapeutic strategies.

Among these novel therapeutic targets are the regulation of unfolded protein response (UPR) pathway, mitochondrial-derived reactive oxygen species (ROS), and specific microRNAs as emerging modulators of plaque biology and stability [75].

Unstable atherosclerotic plaques are the primary cause of fatal outcomes; therefore, there is an urgent need to identify therapies that stabilize plaques to prevent cardiovascular and cerebrovascular diseases. During plaque formation, excessive protein synthesis and misfolding occur, which trigger endoplasmic reticulum (ER) stress [76]. The Unfolded Protein Response (UPR) pathway is chronically activated in atherosclerosis, and while it is initially a protective response, its prolonged activation contributes to the progression of the disease. Persistent ER stress induced by oxidized lipids and inflammatory stimuli promotes apoptosis of macrophages and endothelial cells within atherosclerotic lesions [77]. This process is closely linked to mitochondrial dysfunction, release of apoptogenic factors, STAT1-mediated pro-apoptotic signaling, and NADPH oxidase–dependent ROS generation [78].

RNA therapeutics are a powerful class of pharmacotherapy agents, capable of gene silencing, editing, and expression modulation. Recent clinical trials for lipid-lowering therapy (LLT) have primarily evaluated two RNA approaches: antisense oligonucleotides (ASO) and small interfering RNA (siRNA) [79]. Most current delivery platforms rely on conjugation of nucleic acid payloads to Gal or GalNAc ligands to enable targeted hepatic uptake [80,81].

miRNAs are small non-coding RNAs that regulate gene expression post-transcriptionally by binding mRNA untranslated regions, resulting in degradation of the mRNA or inhibition of translation. Karere et al. [82] aimed to compare miRNA-related mechanisms linked to CVD in early atherosclerotic lesions with healthy sites of arteries and identify gene targets of these miRNAs as well as miRNA-gene networks involved. And indeed, they identified miRNAs expressed in lesions and in blood that correlate with lesion burden and are potential therapeutic targets and biomarkers. These findings support a dual role for miRNAs as both therapeutic targets and circulating biomarkers.

One miRNA of interest may be, for example, miR-26b. With a five-fold increase, miR-26b is highly expressed in human atherosclerotic plaques compared to healthy vessels, thus may have a protective effect [83]. And indeed, the results of Peters et al. [84] clearly demonstrate an atheroprotective role of miR-26b by attenuating lesion formation, mainly by suppressing inflammation and stimulating collagen breakdown. It has been shown that nonhematopoietic-specific deficiency of miR-26b aggravated atherosclerosis, leading to larger plaques with increased collagen deposition and expanded necrotic cores. This was accompanied by elevated expression of vascular cell adhesion molecule-1 (VCAM-1) and increased leukocyte adhesion, as demonstrated in ex vivo perfusion assays. Conversely, restoring miR-26b expression in human coronary artery endothelial cells attenuated inflammatory responses and reduced leukocyte adhesion [84]. Collectively, these data position miR-26b as a promising candidate for therapeutic modulation of plaque stability.

The increasing demand to identify and characterize miRNA–target interactions (MTIs) has driven the development of numerous online resources, including specialized databases and analytical tools. TarBase provides manually curated, experimentally validated MTIs and, in its latest release (v10), also incorporates virally encoded miRNAs [83]. HMDD is a continuously updated repository of experimentally supported miRNA–disease associations derived from the biomedical literature, currently comprising more than 53,000 associations involving almost 1900 miRNAs and 2360 diseases [85]. miRNATissueAtlas2 offers a comprehensive miRNA expression atlas based on 188 tissue samples spanning 21 organ types collected from six individuals. TheMarker is a recently developed, comprehensive database that catalogs diverse therapeutic and monitoring biomarkers, including microRNAs [86]. In addition, extensive collections of miRNA-related single-nucleotide polymorphisms (SNPs) and disease-related variants (DRVs) are available through databases such as dbSNP [87], the GWAS Catalog [88], ClinVar [89], and COSMIC [90].

Despite their promise, the development and clinical translation of miRNA-based therapeutics face substantial regulatory and manufacturing challenges. Regulatory agencies require extensive evidence of safety, efficacy, and target specificity, particularly given the potential for off-target effects due to the pleiotropic nature of miRNA regulation [91]. These challenges currently limit widespread clinical implementation despite strong preclinical rationale.

In parallel with RNA-based strategies, proprotein convertase subtilisin/kexin type 9 (PCSK9) has emerged as a major therapeutic target in lipid metabolism. Fully humanized monoclonal antibodies, including alirocumab and evolocumab, are approved to inhibit PCSK9 activity [92]. The FOURIER trial demonstrated that evolocumab significantly lowered LDL cholesterol and reduced cardiovascular events without major safety concerns [93], while the ODYSSEY OUTCOMES trial showed that alirocumab reduced major adverse cardiovascular events, particularly in patients with higher baseline LDL cholesterol levels [94].

More recently, inclisiran, a small interfering RNA targeting PCSK9 synthesis, has been approved in both Europe and the United States [95]. Inclisiran reduces LDL receptor degradation by silencing PCSK9 mRNA in hepatocytes, resulting in sustained LDL-C lowering. Clinical outcomes from the ORION/VICTORION trial program demonstrate consistent efficacy and favorable tolerability across diverse patient populations [96]. The ORION-13 trial further confirmed the safety and efficacy of inclisiran in adolescents with homozygous familial hypercholesterolemia [97].

Finally, advances in interventional and surgical revascularization strategies—including next-generation stents, multi-arterial bypass grafting, and engineered vascular conduits—have complemented pharmacological progress. Enhanced imaging modalities now support improved lesion characterization and procedural planning, reinforcing an integrated approach to the management of advanced atherosclerotic [98]. Together, these emerging therapies and technologies underscore a shift toward mechanism-driven, plaque-focused intervention aimed at improving long-term cardiovascular outcomes.

## 9. Conclusions

Atherosclerosis remains a complex, multifactorial disease in which structural, cellular, and molecular processes converge to determine plaque behavior and clinical outcome. From a pathological perspective, the disease cannot be adequately understood through luminal narrowing alone; rather, it represents a dynamic interaction between endothelial dysfunction, lipid retention and modification, immune activation, smooth muscle cell phenotypic modulation, extracellular matrix remodeling, and thrombogenic surface alterations [7,12,20,28,33]. These processes evolve heterogeneously across vascular territories and individuals, giving rise to plaques with distinct biological activity and variable propensity for destabilization.

Histopathological classification systems, particularly the AHA framework and its Virmani-modified refinements, have provided an essential foundation for understanding lesion progression and the substrates of acute atherothrombotic events [8,9,10,11,14,34]. Pathology has unequivocally demonstrated that plaque instability is driven by qualitative features—such as fibrous cap thinning, necrotic core expansion, macrophage-dominated inflammation, erosion, and calcified nodules—rather than plaque size alone [3,10,12,32,33,59]. These insights continue to underpin contemporary concepts of risk stratification and inform both imaging interpretation and therapeutic development.

Advances in immunohistochemistry and molecular pathology have further refined our understanding of plaque biology. Beyond identifying cellular composition, modern pathological approaches increasingly capture functional states of macrophages, smooth muscle cells, and extracellular matrix components that govern plaque stability and thrombogenicity [3,10,12,13,30,31,33,99]. The integration of lineage markers with emerging indicators of inflammatory activity, matrix integrity, osteogenic signaling, and local anticoagulant balance underscores the evolving role of pathology in defining biologically meaningful plaque phenotypes.

Importantly, pathology serves as a critical bridge between experimental discoveries, clinical imaging, and therapeutic intervention. Novel pharmacological strategies—including intensive lipid lowering, anti-inflammatory therapies, and RNA-based approaches—ultimately exert their benefit by modifying plaque composition and biology, effects that are most directly validated at the tissue level [3,71,79,93,94,95,96,97,98,100]. Similarly, advances in imaging and functional vascular testing gain interpretative power when grounded in well-characterized histopathological correlates [61,64,65,66,67,68].

In conclusion, pathology remains central to advancing the understanding of atherosclerosis as a biologically active disease and to refining concepts of plaque instability. Future progress will depend on closer integration of digital pathology, quantitative morphometry, molecular profiling, and clinical data to identify actionable plaque phenotypes. From a pathologist’s perspective, the goal is not merely to describe lesions, but to contribute to precision prevention and targeted therapy by defining the structural and biological features that truly drive cardiovascular risk.

## Figures and Tables

**Table 1:** Comparison of AHA classification system and Virmani modified clasification system of atherosclerosis.

Pathological Stage/Feature	AHA Classification	Virmani Classification	Key Histopathological Characteristics
Diffuse intimal response	Type I (initial lesion)/Adaptive intimal thickening	Adaptive intimal thickening	Proteoglycan-rich intima, scattered SMCs, minimal lipid, absence of macrophage foam cells
Foam cell accumulation	Type II (fatty streak)	Intimal xanthoma	Macrophage-derived foam cells with intracellular lipid; preserved intimal architecture
Early extracellular lipid	Type III (intermediate lesion)	Pathological intimal thickening (PIT)	Pools of extracellular lipid without a necrotic core; SMC-rich intima
Established necrotic core	Type IV (atheroma)	Fibroatheroma	Well-formed necrotic lipid core with macrophages and SMCs; variable fibrous cap
Thick fibrous cap plaque	Type V (fibroatheroma)	Thick-cap fibroatheroma	Prominent collagen-rich fibrous cap; smaller necrotic core
Thin fibrous cap	Not distinctly defined	Thin-cap fibroatheroma (TCFA)	Large necrotic core, thin fibrous cap (<65 μm), macrophage infiltration
Plaque rupture	Type VI (complicated lesion)	Plaque rupture	Fibrous cap disruption with luminal thrombosis
Plaque erosion	Included within Type VI	Plaque erosion	Endothelial denudation without cap rupture; proteoglycan-rich surface
Calcified nodules	Included within Type VI	Calcified nodule	Protruding calcific masses with disrupted fibrous cap
Predominantly fibrotic plaque	Type VIII (fibrotic lesion)	Fibrotic plaque	Dense collagen, minimal lipid, reduced inflammation

## Data Availability

All data discussed in this review are derived from previously published studies, which are cited in the reference list.

## References

[1] Roth G.A., Mensah G.A., Johnson C.O. (2023). Global burden of cardiovascular diseases and risk factors, 1990–2019: Update from the GBD 2019 Study. J. Am. Coll. Cardiol..

[2] World Health Organization (2023). Cardiovascular Diseases (CVDs): Key Facts.

[3] Libby P., Buring J.E., Badimon L. (2023). Atherosclerosis. Nat. Rev. Dis. Primers.

[4] Hansson G.K., Hermansson A. (2023). The immune system in atherosclerosis. Nat. Immunol..

[5] Ross R. (1999). Atherosclerosis—An inflammatory disease. N. Engl. J. Med..

[6] Gimbrone M.A., Garcia-Cardena G. (2016). Endothelial cell dysfunction and the pathobiology of atherosclerosis. Circ. Res..

[7] Lusis A.J. (2000). Atherosclerosis. Nature.

[8] Stary H.C., Chandler A.B., Glagov S. (1994). A definition of initial, fatty streak, and intermediate lesions of atherosclerosis. Circulation.

[9] Stary H.C., Chandler A.B., Dinsmore R.E. (1995). A definition of advanced types of atherosclerotic lesions. Circulation.

[10] Virmani R., Kolodgie F.D., Burke A.P., Farb A., Schwartz S.M. (2000). Lessons from sudden coronary death: A comprehensive morphological classification scheme. Arterioscler. Thromb. Vasc. Biol..

[11] Pasterkamp G., den Ruijter H.M. (2022). Pathology of atherosclerotic plaque progression and vulnerability. Cardiovasc. Pathol..

[12] Davies M.J. (1996). Stability and instability: Two faces of coronary atherosclerosis: The Paul Dudley White Lecture 1995. Circulation.

[13] Libby P., Hansson G.K. (2015). Inflammation and immunity in diseases of the arterial tree: Players and layers. Circ. Res..

[14] Otsuka F., Yasuda S., Noguchi T., Ishibashi-Ueda H. (2023). Pathology of coronary atherosclerosis and thrombosis. Cardiovasc. Diagn. Ther..

[15] Bennett M.R., Sinha S., Owens G.K. (2022). Vascular smooth muscle cells in atherosclerosis. Circ. Res..

[16] Wirka R.C. (2023). Smooth muscle cell phenotypic switching in human atherosclerosis. Nat. Med..

[17] Fernandez D.M. (2024). Single-cell immune profiling of human atherosclerotic plaques. Circulation.

[18] Saigusa R., Winkels H., Ley K. (2022). T cell subsets and functions in atherosclerosis. Nat. Rev. Cardiol..

[19] Holm N.R. (2023). Extracellular matrix remodeling and fibrous cap weakening in advanced atherosclerosis. Atherosclerosis.

[20] Yusuf S., Joseph P., Rangarajan S. (2022). Modifiable risk factors, cardiovascular disease, and mortality in 155,722 individuals from 21 countries (PURE study). Lancet.

[21] Herrington W., Lacey B., Sherliker P., Armitage J., Lewington S. (2023). Epidemiology of atherosclerotic cardiovascular disease and risk factors. Nat. Rev. Cardiol..

[22] Rit E. (2004). Atherosclerosis in dialyzed patients. Blood Purif..

[23] Davis C., Fischer J., Ley K., Sarembock I.J. (2003). The role of inflammation in vascular injury and repair. J. Thromb. Haemost..

[24] Savinov A.Y. (2015). Transgenic overexpression of tissue-nonspecific alkaline phosphatase in vascular endothelium results in generalized arterial calcification. J. Am. Heart Assoc..

[25] Maiellaro K., Taylor W.R. (2007). The role of the adventitia in vascular inflammation. Cardiovasc. Res..

[26] Melaku L., Addisu D. (2021). The cellular biology of atherosclerosis with atherosclerotic lesion classification and biomarkers. Bull. Natl. Res. Cent..

[27] Steinberg D., Witztum J.L. (2010). Oxidized low-density lipoprotein and atherosclerosis. Arterioscler. Thromb. Vasc. Biol..

[28] Jebari-Benslaiman S. (2022). Pathophysiology of atherosclerosis. Int. J. Mol. Sci..

[29] Moore K.J., Tabas I. (2011). Macrophages in the pathogenesis of atherosclerosis. Cell.

[30] Francis G.A. (2023). The greatly under-represented role of smooth muscle cells in atherosclerosis. Curr. Atheroscler. Rep..

[31] Tabas I., Bornfeldt K.E. (2020). Intracellular and intercellular aspects of macrophage immunometabolism in atherosclerosis. Circ. Res..

[32] Xepapadaki E. (2020). The antioxidant function of HDL in atherosclerosis. Angiology.

[33] Bentzon J.F., Otsuka F., Virmani R., Falk E. (2024). Mechanisms of plaque formation and rupture. Circ. Res..

[34] Daugherty A., Tall A.R., Daemen M., Falk E., Fisher E.A., García-Cardeña G., Lusis A.J., Owens A.P., Rosenfeld M.E., Virmani R. (2017). Recommendation on Design, Execution, and Reporting of Animal Atherosclerosis Studies: A Scientific Statement From the American Heart Association. Arter. Thromb. Vasc. Biol..

[35] van Veelen A., Sangen N., Henriques J., Claessen B. (2022). Identification and treatment of the vulnerable coronary plaque. Rev. Cardiovasc. Med..

[36] Konishi T., Funayama N., Virmani R. (2023). Pathological characteristics of macrophage-rich coronary plaques associated with thrombosis. Cardiovasc. Pathol..

[37] Balmos I.A. (2023). Macrophage infiltration and fibrous cap thinning in advanced carotid atherosclerosis. Atherosclerosis.

[38] Gisterå A. (2024). B cells and tertiary lymphoid structures in atherosclerosis. Eur. Heart J..

[39] Cochain C. (2018). Single-cell RNA-seq reveals the transcriptional landscape and heterogeneity of aortic macrophages in atherosclerosis. Nat. Commun..

[40] Kim K., Shim D., Lee J.S. (2023). Transcriptome analysis reveals TREM2-positive macrophages associated with lipid metabolism and plaque progression in human atherosclerosis. Circulation.

[41] Tasheva E.S., Koester A., Paulsen A.Q. (2002). Mimecan/osteoglycin regulates collagen fibrillogenesis in connective tissues. J. Biol. Chem..

[42] Perisic Matic L. (2022). Osteoglycin deficiency leads to altered collagen organization and increased plaque vulnerability in human atherosclerosis. Cardiovasc. Res..

[43] Brandenburg V.M., Kramann R. (2019). Calcification of the vascular system: The skeleton in the vessel wall. Circ. Res..

[44] Durham A.L., Speer M.Y., Scatena M. (2018). Role of smooth muscle cells in vascular calcification. Arterioscler. Thromb. Vasc. Biol..

[45] Evenepoel P., D’Haese P., Brandenburg V. (2023). Sclerostin and vascular calcification: Emerging concepts. Kidney Int..

[46] Qureshi A.R. (2024). Sclerostin expression in calcified atherosclerotic plaques and its association with cardiovascular outcomes. Atherosclerosis.

[47] Matsuda M., Shimomura I., Sata M. (2002). Role of adiponectin in preventing vascular inflammation. Circulation.

[48] Okamoto Y., Kihara S., Funahashi T. (2008). Adiponectin: A key adipocytokine in metabolic syndrome. Arterioscler. Thromb. Vasc. Biol..

[49] Ouchi N., Walsh K. (2022). Adiponectin as an anti-inflammatory factor in vascular disease. Nat. Rev. Cardiol..

[50] Yamauchi T., Kadowaki T. (2023). Adiponectin signaling and cardiovascular protection. Circulation.

[51] Hinson J.P., Kapas S., Smith D.M. (2000). Adrenomedullin, a multifunctional regulatory peptide. Circ. Res..

[52] Kawai J., Ando K., Horiuchi M. (2004). Adrenomedullin inhibits apoptosis in endothelial cells. Arterioscler. Thromb. Vasc. Biol..

[53] Kitamura K., Eto T. (2022). Adrenomedullin and cardiovascular disease. Physiol. Rev..

[54] Koyama T. (2023). Adrenomedullin expression in atherosclerotic lesions and vascular stress responses. Atherosclerosis.

[55] Blinder M.A., Marasa J.C., Reynolds C.H. (2002). Dermatan sulfate-dependent inhibition of thrombin by heparin cofactor II in the arterial wall. J. Clin. Investig..

[56] Raghavan S., Singh N., Fogelson A.L. (2008). Localization of heparin cofactor II in human atherosclerotic plaques. Arterioscler. Thromb. Vasc. Biol..

[57] Takamura M. (2024). Local anticoagulant mechanisms in the arterial wall: Role of heparin cofactor II. Thromb. Haemost..

[58] Tolle M. (2022). Heparin cofactor II and vascular protection in atherosclerosis. Cardiovasc. Res..

[59] Virmani R., Burke A.P., Farb A., Kolodgie F.D. (2006). Pathology of the vulnerable plaque. J. Am. Coll. Cardiol..

[60] Goff D.C. (2014). 2013 ACC/AHA guideline on the assessment of cardiovascular risk: A report of the American College of Cardiology/American Heart Association Task Force on Practice Guidelines. Circulation.

[61] Pleitez M.A. (2020). Label-free metabolic imaging by mid-infrared optoacoustic microscopy in living cells. Nat. Biotechnol..

[62] Greenland P. (2018). Coronary calcium score and cardiovascular risk. J. Am. Coll. Cardiol..

[63] Mach F., Baigent C., Catapano A.L., Koskinas K.C., Casula M. (2020). 2019 ESC/EAS Guidelines for the management of dyslipidaemias: Lipid modification to reduce cardiovascular risk. Eur. Heart J..

[64] Thijssen D.H.J., Bruno R.M., van Mil A. (2022). Expert consensus and evidence on the assessment of flow-mediated dilation. Eur. Heart J..

[65] Ghiadoni L., Taddei S., Virdis A. (2023). Endothelial function and cardiovascular disease. Nat. Rev. Cardiol..

[66] Laurent S., Boutouyrie P. (2022). Arterial stiffness and cardiovascular risk. Nat. Rev. Cardiol..

[67] Townsend R.R., Wilkinson I.B., Schiffrin E.L. (2023). Recommendations for improving and standardizing vascular research on arterial stiffness. Hypertension.

[68] Cecelja M., Chowienczyk P. (2024). Role of arterial stiffness in atherosclerosis and cardiovascular risk. Cardiovasc. Res..

[69] World Health Organization (2020). WHO Guidelines on Physical Activity and Sedentary Behaviour.

[70] Visseren F.L.J., Mach F., Smulders Y.M., Carballo D., Koskinas K.C. (2021). 2021 ESC Guidelines on cardiovascular disease prevention in clinical practice. Eur. Heart J..

[71] Steinberg D. (2009). The LDL modification hypothesis of atherogenesis: An update. J. Lipid Res..

[72] Williams B., Mancia G., Spiering W., Agabiti Rosei E., Azizi M. (2018). 2018 ESC/ESH Guidelines for the management of arterial hypertension. Eur. Heart J..

[73] Weber C., Noels H. (2011). Atherosclerosis: Current pathogenesis and therapeutic options. Nat. Med..

[74] McEvoy J.W. (2023). 2023 ESC guidelines for the management of elevated blood pressure and hypertension. Eur. Heart J..

[75] Liu L., Wang Z., Ma L., Wu S. (2025). Recent insights into the pathophysiology and implications for surgery in atherosclerosis. J. Cardiothorac. Surg..

[76] Niu H., Wu L., Cai Y., Yu C., Lin N., Cai X., Chen M., Wang L. (2025). Endoplasmic reticulum-associated degradation mitigates atherosclerosis by maintaining cellular homeostasis. Front. Physiol..

[77] Zhou A.X., Tabas I. (2013). The UPR in atherosclerosis. Semin. Immunopathol..

[78] Ghosh A.P., Klocke B.J., Ballestas M.E., Roth K.A. (2012). CHOP potentially cooperates with FOXO3a in neuronal cells to regulate PUMA and BIM expression in response to ER stress. PLoS ONE.

[79] Maidman S.D., Rosenson R.S. (2025). Lipid-lowering RNA therapeutics for atherosclerotic cardiovascular disease prevention. BioDrugs.

[80] Androsavich J.R. (2024). Frameworks for transformational breakthroughs in RNA-based medicines. Nat. Rev. Drug Discov..

[81] Ramirez-Cortes F., Menova P. (2024). Hepatocyte targeting via the asialoglycoprotein receptor. RSC Med. Chem..

[82] Karere G.M. (2023). Potential miRNA biomarkers and therapeutic targets for early atherosclerotic lesions. Sci. Rep..

[83] Skoufos G., Kakoulidis P., Tastsoglou S. (2024). TarBase v9.0 extends experimentally supported miRNA-gene interactions to cell types and virally encoded miRNAs. Nucleic Acids Res..

[84] Peters L.J.F., Bidzhekov K., Bonnin-Marquez A. (2025). MicroRNA-26b deficiency augments atherosclerosis, while mimic-loaded nanoparticles reduce atherogenesis. Cardiovasc. Res..

[85] Cui C., Zhong B., Fan R., Cui Q. (2024). HMDD v4.0: A database for experimentally supported human microRNA-disease associations. Nucleic Acids Res..

[86] Zhang Y. (2024). TheMarker: A comprehensive database of therapeutic biomarkers. Nucleic Acids Res..

[87] Sherry S.T., Ward M.H., Kholodov M. (2001). dbSNP: The NCBI database of genetic variation. Nucleic Acids Res..

[88] Buniello A., MacArthur J.A.L., Cerezo M. (2019). The NHGRI-EBI GWAS Catalog of published genome-wide association studies. Nucleic Acids Res..

[89] Landrum M.J., Chitipiralla S., Brown G.R. (2020). ClinVar: Improvements to accessing data. Nucleic Acids Res..

[90] Tate J.G., Bamford S., Jubb H.C. (2019). COSMIC: The Catalogue of Somatic Mutations in Cancer. Nucleic Acids Res..

[91] Chakraborty C., Sharma A.R., Sharma G., Lee S.S. (2020). Therapeutic advances of miRNAs: A preclinical and clinical update. J. Adv. Res..

[92] Pokhrel B., Pellegrini M.V., Levine S.N. (2025). PCSK9 Inhibitors.

[93] Sabatine M.S., Giugliano R.P., Keech A.C. (2017). Evolocumab and clinical outcomes in patients with cardiovascular disease. N. Engl. J. Med..

[94] Schwartz G.G., Steg P.G., Szarek M. (2018). Alirocumab and cardiovascular outcomes after acute coronary syndrome. N. Engl. J. Med..

[95] Marrs J.C., Anderson S.L. (2024). Inclisiran for the treatment of hypercholesterolaemia. Drugs Context.

[96] Katsiki N., Vrablik M., Banach M., Gouni-Berthold I. (2023). Inclisiran, low-density lipoprotein cholesterol and lipoprotein(a). Pharmaceuticals.

[97] Wiegman A., Peterson A.L., Hegele R.A. (2025). Efficacy and safety of inclisiran in adolescents with homozygous familial hypercholesterolemia. Circulation.

[98] Syed M.B. (2019). Emerging techniques in atherosclerosis imaging. Br. J. Radiol..

[99] Puz P. (2013). Inflammatory markers in patients with internal carotid artery stenosis. Arch. Med. Sci..

[100] Orekhov A.N. (2013). Mitochondrion as a selective target for treatment of atherosclerosis. Curr. Neuropharmacol..

